# Evidence-Based Practice in Psychology Fails to Be Tripartite: A Conceptual Critique of the Scientocentrism in Evidence-Based Practice in Psychology

**DOI:** 10.3389/fpsyg.2019.02253

**Published:** 2019-10-09

**Authors:** Henrik Berg

**Affiliations:** Department of Education, NLA University College, Bergen, Norway

**Keywords:** evidence-based practice in psychology, psychotherapy science, science and practice, critique, clinical expertise, patient values

## Abstract

This paper criticises evidence-based practice in psychology (EBPP) for not actually being a tripartite model. According to the American Psychological Association, EBPP is defined as the integration of the best available research with clinical expertise in the context of patient characteristics, culture, and preferences. Nonetheless, EBPP fails to be a tripartite model because it is defined by science alone. This paper aims at explaining why this conflation may have come about. It also shows why clinical expertise and patient preferences should be defined extra-scientifically.

## Introduction

The policy statement for evidence-based practice in psychology (EBPP) is the prevailing set of principles regulating psychotherapy practice (American Psychological Association, 2016). The American psychological association (APA) defines EBPP as: “the integration of the best available research with clinical expertise in the context of patient characteristics, culture, and preferences” (Levant, 2005). According to this definition, EBPP consists of three distinct parts (“best available research”; “clinical expertise”; and “patient characteristics, culture, and preferences”). “Best available research” refers to the body of scientific evidence underpinning various psychological interventions. “Clinical expertise” is the competency of the clinical expert responsible for carrying out the interventions. “Patient characteristics, culture, and preferences” refer to the most relevant aspects distinguishing individual patients.

In EBPP, these three parts are supposed to be integrated to create best psychotherapy practice. However, the policy statement contains a crucial inconsistency. EBPP does not consist of three parts. The two parts, clinical expertise and patient characteristics, culture, and preferences, are legitimated and shaped by the part “best available research” (American Psychological Association, 2016). Consequently, important extra-scientific reasons for including clinical expertise and patient characteristics, culture, and preferences in clinical practice are neglected.

### Lost in Translation: From EBM to EBPP

A recapitulation of EBPP’s history is helpful to shed some light on why these issues may have gone unnoticed. EBPP explicitly derived from evidence-based medicine (EBM). The seminal pioneers in EBM – predating both the current tripartite model in EBM and the subsequent tripartite model in EBPP – simply maintained that medical practice ought to correspond to the results of randomised controlled trials and meta-analyses (consisting of compilations of randomised controlled trials) (Cochrane, 1999; Howick, 2011). Thus, when the tripartite model in EBM was created, it was a constructive response to criticisms of a “scientocentric” model of medical practice in the original EBM models. After some revision, two additional parts were suggested. In the first tripartite EBM model, these parts were “clinical expertise” and “patient values.” Hence, the rationale for creating the tripartite version of EBM, on which EBPP is modelled, was to avoid and not to corroborate “scientocentrism” (Howick, 2011).

#### “Scientocentrism” in EBPP

In spite of its historical background, EBPP’s tripartite model is justified scientifically through the part called “best available research” (Norcross et al., 2008; Gupta, 2014; American Psychological Association, 2016). In the policy-statement, the two parts called “clinical expertise” and “patient characteristics, culture, and preferences” are legitimated and defined through empirical studies indicating that they are significant factors in effective and efficient psychotherapy (American Psychological Association, 2016). In the policy statement it is argued that “[c]linical expertise encompasses a number of competencies” that are known to “promote positive therapeutic outcomes” (Levant, 2005). As expected, the definition of patient characteristics, culture, and preferences is comprehensive and includes elements such as religious views, personality traits, and personal preferences. Nonetheless, patient preferences is a legitimate part because an understanding of “‘what works for whom’ […] provide[s] essential guide to effective practice.” (Levant, 2005). Thus, on closer scrutiny, the purportedly tripartite model in EBPP collapses into a unipartite and “scientocentric” model.

#### Medicine and Psychotherapy

Some of the differences between medical and psychotherapy practice can explain the conceptual confusion in EBPP. If they were applied to medical practice, the three parts in EBPP would be more readily discernible. Many medical cures are effective regardless of the involvement of a clinical expert and without emphasising patient characteristics, culture, or preferences in the treatment process. Furthermore, medical clinical expertise includes elements such as the craftsmanship of the medical doctor (e.g., the dexterity of a surgeon). In psychotherapy, however, some of the major factors empirically associated with good psychotherapy are tied to clinical expertise and patient culture and characteristics (Lambert and Barley, 2001; Lambert and Vermeersch, 2002; Norcross, 2002; Wampold, 2015; Asarnow and Ougrin, 2017; Mulder et al., 2017; Zilcha-Mano, 2017). Wampold (2015) has shown that factors such as goal consensus/collaboration, empathy, the alliance, positive regard/affirmation, therapist effects, and congruence/genuineness outweigh the importance of differences in specific treatment models. However, research also indicate that expertise is only associated with better outcome under specific circumstances (Kahneman and Klein, 2009). There is similar research evidence supporting the importance of patient culture and characteristics (Lambert and Vermeersch, 2002). Lambert and Vermeersch (2002) argue that “it is […] the patient who is most likely to determine successful outcome.” The authors go on to claim that “[p]atient variables found to have a relationship with outcome are severity of disturbance, motivation and expectancy, capacity to relate, degree of integration, coherence, perfectionism, and ability to recognize and verbalize focalized problems” (Lambert and Vermeersch, 2002). In addition, cultural adaptation is associated with improved outcomes. It explains more of the variation in outcome than choice of treatment method (Wampold, 2015). Apparently, such research findings have contributed to a conflation of the two parts clinical expertise and patient characteristics, culture, and preferences, on the one hand, and the best available research indicating that the clinical expert and patient culture and preferences play a major part in clinical effectiveness and efficiency, on the other hand.

#### From One to Three Parts

In order to rehabilitate EBPP, its history could prove useful once more. This especially pertains to the expansion of EBM into a tripartite model (Howick, 2011). A first and critical point is to make a conceptual distinction between factors empirically associated with effective and efficient psychotherapy, on the one hand, and conceptual and practical issues caused by the void between scientific findings and the complexities of clinical practice, on the other hand. More precisely, it must be shown how “clinical expertise” and “patient characteristics, culture, and preferences” differ from “best available research.” The role clinical expertise and patient characteristics, culture, and preferences play in psychotherapy should nevertheless be investigated empirically. Such empirical analyses potentially have immense clinical value (Appelbaum et al., 2018; Levitt et al., 2018). Nevertheless, the rationales for including and the definition of, the two parts “clinical expertise” and “patient culture, characteristics, and preferences,” must transcend the limits of science (Powell, 2018).

It is worth mentioning that there are other available alternatives than going from a unipartite to a genuinely tripartite concept. The policy statement of psychological treatments created by the Canadian Psychological Association is unipartite and emphasises scientific findings in every aspect of psychological treatment (Canadian Psychological Association, 2012). The advantage of this approach is that the demarcation of the conception is clearer. The disadvantage is that it could preclude the importance of elements that are non-scientific. Due to the risk of the latter, I pursue the option of restoring EBPP in line with the primordial intensions of creating the concept.

#### Clinical Expertise

In the policy statement for EBPP, scientific findings define clinical expertise and the ideal clinical expert emulate a scientist. The ideal EBPP practitioner “test[s] hypotheses and interventions in practice as a ‘local clinical scientist”’ (American Psychological Association, 2016). In the policy statement, it is recognised that clinical experts process information differently from novices and that clinical practice corroborates expertise. Nonetheless, it emphasises how experts seek and use scientific knowledge in a manner emulating scientific practice (American Psychological Association, 2016).

In contrast, some of the most influential models of expertise accentuates that one of its hallmarks is that it transcends propositional or scientific knowledge (Ryle, 1945; Heidegger, 1962; Dreyfus and Dreyfus, 1980; Schön, 1991; Polanyi, 2009; Greenwald and Banaji, 2017). The experience of the expert results in a greater number of relatively refined templates from which to understand and act (Dreyfus and Dreyfus, 1980). When necessary, the practical experience of the expert provides a greater ability to improvise and synthesise information in novel situations (Schön, 1991). The point is not that the understanding and actions of the expert need to be at odds with scientific results, but that the understanding and actions of the expert are not perpetually controlled by scientific or propositional knowledge (Ryle, 1945; Selinger and Crease, 2002). Whereas the novice is fragmentally rule-bound, the expert acts more intuitively (Dreyfus and Dreyfus, 1980; Selinger and Crease, 2002). Furthermore, the expert has a deep and often at least partly tacit knowledge (Polanyi, 2009), including a rich situational and contextual awareness (Heidegger, 1962; Dreyfus and Dreyfus, 1980; Schön, 1991; Galasiński, 2018). *In situ*, this results in a capability to handle the many arising challenges and impediments with the fluency generally demanded in settings such as psychotherapy practice.

#### Patient Characteristics, Culture, and Preferences

In the policy statement, it is argued that there is a “growing empirical literature related to human diversity” which makes “an understanding of patient characteristics” like “values, religious beliefs, worldviews, goals, and preferences […] essential to EBPP” (American Psychological Association, 2016). In other words, the justification for including and the definition of the part patient characteristics, culture, and preferences are scientific. However, a critical extra-scientific argument for including the part “patient characteristics, culture, and preferences” is that the patient should have the right to influence choices involving the patient’s own life. This is highlighted by the term “preferences” in the policy statement, notwithstanding, of course, that preferences are entangled with characteristics and culture.

Patients do and should have extensive right to self-determination (United Nations, 1991; Pellegrino and Thomasma, 1993; Leong et al., 2017). As an example, one of the five general principles in the ethical principles for psychologists and code of conduct (EPPCC) (American Psychological Association, 2017) is “the rights of individuals to [have] […] self-determination” (American Psychological Association, 2017). In other words, the inclusion of “patient preferences” is an end in itself and not merely a means to other ends (i.e., improved efficiency or efficacy). This also entails that patient preferences ought to play a significant role in clinical practice even when the patient prefers something that diverges from what science indicates would be, or even *de facto* is, effective and efficient. The individual patient’s preferences are not determined by scientific findings.

The scientocentrism addressed in this article reflects a growing recognition of the problems associated with EBPP. Several scholars have argued that there is a science-practice gap in psychotherapy which makes a scientocentric model of psychotherapy practice unfeasible (Lilienfeld et al., 2013; Cook et al., 2017). Hayes et al. (2013) claimed that psychological research is theory-driven and that there are many different scientific schools valuing different kinds of descriptions. Berg and Slaattelid (2017) argued that EBPP fails to recognise the ethoses of the various psychotherapy schools. An ethos consists of a psychotherapy school’s normative presuppositions. Berg (2019) also argued that EBPP functions as an implicit ethical demarcation regulating the practice of psychotherapy according to the tenets of utilitarianism. Generally speaking the role values and ethics plays in psychotherapy practice necessitate the active deliberation of a clinical expert and a patient. The deep fact-value entanglements and complexity of psychotherapy make a scientocentric model unfit to regulate psychotherapy practice.

## Conclusion

The policy-statement should be revised to avoid the conceptual inconsistency it currently contains. The solution is to create a genuinely tripartite model to replace the current “scientocentric” one. Future research should discuss the nature of and relationship between the different parts of EBPP. It should also develop the notion of integration which has been characterised as underdeveloped. In a practice as complex as psychotherapy we should expect on-going revisions of the principles regulating the practice and not settle for a solution lacking conceptual consistency (Jackson, 2017; Phelps et al., 2017).

## Author Contributions

The author confirms being the sole contributor of this work and has approved it for publication.

## Conflict of Interest

The authors declare that the research was conducted in the absence of any commercial or financial relationships that could be construed as a potential conflict of interest.
